# Frequency and Characteristics of Gastrointestinal Diseases in Patients With Neurofibromatosis

**DOI:** 10.1002/jgh3.70151

**Published:** 2025-04-16

**Authors:** Manami Honda, Masaya Iwamuro, Takehiro Tanaka, Yasushi Yamasaki, Seiji Kawano, Sakiko Hiraoka, Yoshiro Kawahara, Motoyuki Otsuka

**Affiliations:** ^1^ Department of Gastroenterology and Hepatology Okayama University Graduate School of Medicine, Dentistry and Pharmaceutical Sciences Okayama Japan; ^2^ Department of Pathology Okayama University Graduate School of Medicine, Dentistry, and Pharmaceutical Sciences Okayama Japan; ^3^ Inflammatory Bowel Disease Center Okayama University Hospital Okayama Japan; ^4^ Department of Practical Gastrointestinal Endoscopy Okayama University Hospital Okayama Japan

**Keywords:** colonoscopy, esophagogastroduodenoscopy, gastrointestinal neoplasms, gastrointestinal stromal tumor, neurofibromatosis

## Abstract

**Background and Aim:**

Patients with neurofibromatosis (NF) frequently experience gastrointestinal symptoms, but the specific characteristics of these lesions are not well understood.

**Methods:**

To investigate the prevalence and nature of gastrointestinal diseases in this population, we analyzed the gastrointestinal lesions identified through endoscopic examinations in patients with NF.

**Results:**

We included 225 patients with NF type 1 (NF1) and 15 with NF type 2 (NF2). None of the NF2 patients underwent endoscopy. Among the NF1 patients, 27 received endoscopies, and 13 (59%) had gastrointestinal lesions. These 13 patients were predominantly male (10 males and three females), with a median age of 53 years (range: 19–76 years). The identified lesions included colorectal polyps (*n* = 6), gastrointestinal stromal tumors ([GIST], *n* = 4), subepithelial lesions (*n* = 3), gastric fundic gland polyps (*n* = 3), diffuse intestinal ganglioneuromatosis (*n* = 2), esophageal polyps (*n* = 2), a Schwann cell hamartoma (*n* = 1), esophageal cancer (*n* = 1), and a gastric hyperplastic polyp (*n* = 1). All GISTs and one case of diffuse intestinal ganglioneuromatosis were surgically resected. Interestingly, six out of 13 patients were asymptomatic. Additionally, all patients who required surgery were 40 years of age or older.

**Conclusions:**

These findings suggest that routine endoscopic examinations, along with imaging techniques like computed tomography and magnetic resonance imaging, could be beneficial for the early detection of gastrointestinal lesions in NF1 patients aged 40 and above.

## 1 Introduction

Neurofibromatosis type 1 (NF1) and type 2 (NF2) are genetic disorders caused by mutations in specific genes. NF1, affecting roughly one in 3000–4000 individuals, is characterized by café‐au‐lait spots, neurofibromas, and Lisch nodules [1, 2, 3]. Patients may also experience freckling in the armpits or groin, skeletal abnormalities like scoliosis and bone dysplasia, and optic pathway gliomas (tumors of the optic nerve). Additionally, NF1 is associated with an increased risk of malignancies such as malignant peripheral nerve sheath tumors and certain leukemias.

In contrast, NF2 is a less common disorder, affecting one in 25 000–40 000 individuals, caused by mutations in a different gene on chromosome 22 [1]. Its hallmark feature is bilateral vestibular schwannomas. Other key features include schwannomas of other cranial and peripheral nerves, meningiomas, ependymomas, and early‐onset cataracts.

While less common than other symptoms, patients with NF, particularly NF1, can experience various gastrointestinal manifestations [4, 5, 6]. These manifestations can lead to serious complications and may require surgery. Understanding the characteristics of these gastrointestinal problems is crucial for accurate diagnosis and effective management. However, due to the relative rarity of gastrointestinal involvement in NF, the incidence and detailed features of these lesions have not been exclusively investigated.

This study aimed to analyze the characteristics of patients with NF and associated gastrointestinal diseases.

## 2 Methods

### 2.1 Patients and Ethics

**FIGURE 1:**
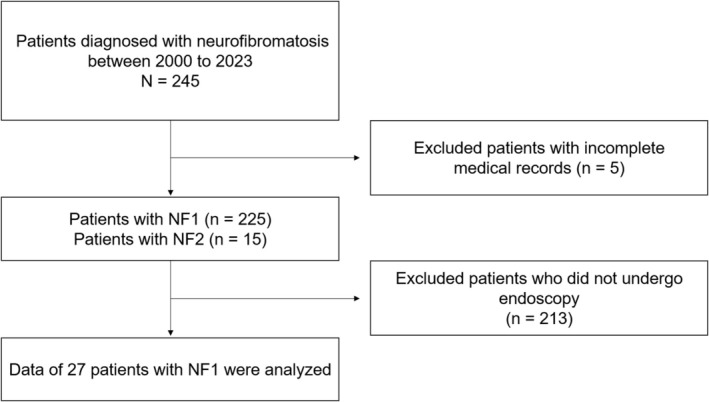
Study flowchart. NF, neurofibromatosis; NF1, neurofibromatosis type 1; NF2, neurofibromatosis type 2.

We retrospectively collected data from patients clinically diagnosed with NF1 or NF2 at the Okayama University Hospital between January 2000 and December 2023 (Figure 1). The diagnosis of NF1 and NF2 was established based on the presence of a pathogenic variant in the *NF1* or *NF2* gene or a combination of clinical features. The clinical diagnostic criteria for NF1 required the presence of at least two out of seven features: (i) ≥ 6 café‐au‐lait spots, (ii) ≥ 2 neurofibromas (cutaneous or plexiform) or one diffuse neurofibroma, (iii) axillary or inguinal freckling, (iv) an optic pathway glioma, (v) ≥ 2 Lisch nodules, (vi) characteristic osseous lesions (such as spinal, thoracic, limb, or craniofacial bone deformities), and (vii) a first‐degree relative with NF1. The clinical diagnostic criteria for NF2 included (i) the presence of bilateral vestibular schwannomas on magnetic resonance imaging (MRI) or computed tomography (CT) or (ii) a history of NF2 in a parent, sibling, or child, along with either (iia) a unilateral vestibular schwannoma or (iib) at least two of the following: schwannomas, meningiomas, gliomas, or juvenile cataracts. Patients with incomplete medical records were excluded. Since this study focused on gastrointestinal lesions, we further excluded those who did not undergo endoscopic examinations, such as esophagogastroduodenoscopy, colonoscopy, balloon‐assisted enteroscopy, video capsule enteroscopy, or endoscopic retrograde cholangiopancreatography. Notably, one patient included in this analysis was previously reported in another publication [7].

This study received approval from the Ethics Committee of Okayama University Hospital. Due to the retrospective nature of the study and the use of anonymized clinical data, the requirement for written informed consent was waived. All procedures complied with relevant guidelines and regulations and adhered to the Declaration of Helsinki.

### 2.2 Immunohistochemistry

Unstained formalin‐embedded tissue samples of gastrointestinal stromal tumors (GISTs) from four patients with NF1 were retrieved from our institution's Department of Pathology. Immunohistochemistry was performed on these samples using the Simple Stain Kit (Nichirei Biosciences, Tokyo, Japan), following the manufacturer's protocol. The primary antibodies employed were anti‐c‐kit (#413391), anti‐CD34 (NU‐4A1, #413111), anti‐S‐100 (#422091), and anti‐desmin (#413651), all obtained from Nichirei Biosciences (Tokyo, Japan). Antigen retrieval was achieved using citrate buffer (pH = 9) in an autoclave. Detection was subsequently performed using Simple Stain DAB solution (#415171, Nichirei Biosciences). Additionally, corresponding tissue sections stained with hematoxylin and eosin were obtained from the Department of Pathology. All stained sections were then analyzed using an AxioObserver 7 microscope (Carl Zeiss, Oberkochen, Germany) and ZEN image analysis software.

### 2.3 Statistics

Statistical analyses were conducted using either the *t* test or *F* test. Statistical significance was set at *p* < 0.05.

## 3 Results

**FIGURE 2:**
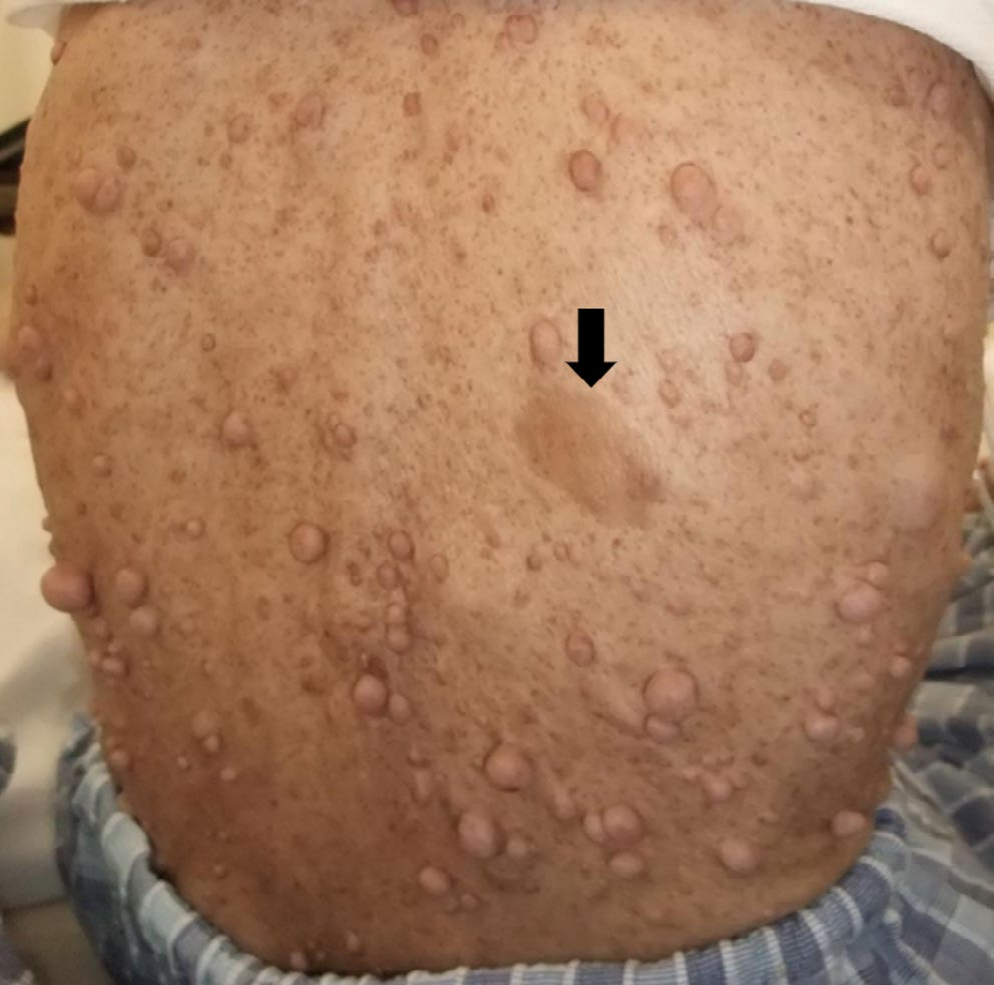
Skin lesions in the back of a patient with NF1. Representative photographs of typical skin lesions observed on the back of a patient with neurofibromatosis type 1. Neurofibromas appear as soft, skin‐colored, or slightly pigmented nodules, varying in size from a few millimeters to several centimeters, and are sessile or pedunculated. A Café‐au‐lait spot, an oval‐shaped pigmented area, is also observed (arrow).

Between January 2000 and December 2023, our institution diagnosed a total of 245 patients with NF. Five patients were excluded due to incomplete medical records. Café‐au‐lait spots and neurofibromas are characteristic findings in patients with NF1 (Figure 2).

Electronic medical records were reviewed to identify which of the 225 patients with NF1 and 15 with NF2 had undergone endoscopic examinations. Regarding the diagnoses of NF1 and NF2, pathogenic variants in the *NF1* and *NF2* genes were identified in 37 patients and one patient, respectively. The remaining 188 and 14 patients were clinically diagnosed with NF1 and NF2, respectively. We excluded 213 patients who did not undergo an endoscopy (Figure 1). The remaining patients underwent various endoscopic procedures, including esophagogastroduodenoscopy (*n* = 22), colonoscopy (*n* = 14), video capsule enteroscopy (*n* = 5), balloon‐assisted enteroscopy (*n* = 3), and endoscopic retrograde cholangiopancreatography (*n* = 3). Additionally, 119 patients received abdominal CT scans. Notably, none of the NF2 patients underwent endoscopy. Finally, this study focused on the 27 patients with NF1 who had undergone endoscopic examinations.

Table 1 summarizes the gastrointestinal lesions detected during endoscopy in 13 of these patients (Group A). The remaining 14 patients with NF1 (Group B) showed no evidence of gastrointestinal lesions. Group A comprised 10 males and three females, with a median age of 53 years (range: 19–76 years) at the time of endoscopy. Group B, which also underwent endoscopic examination, consisted of seven males and seven females, with a median age of 33.5 years (range: 18–70 years). Patients in Group A were significantly older than those in Group B (*p* = 0.015). However, there was no statistically significant difference in sex distribution between the groups (*p* = 0.237).

**TABLE 1:** Gastrointestinal lesions detected in patients with neurofibromatosis type 1.

Case no.	Age	Sex	Symptoms	Gastrointestinal lesions
1	53	M	None	Gastric GIST, gastric fundic gland polyp, colorectal polyp
2	44	M	GI bleeding	Duodenal GIST, gastric fundic gland polyp
3	54	M	GI bleeding	Jejunal GIST, esophageal SEL, colorectal polyp
4	76	M	GI bleeding	Jejunal and ileal GIST
5	57	M	Abdominal pain	Colorectal diffuse intestinal ganglioneuromatosis, gastric SEL, esophageal cancer
6	27	M	None	Rectal diffuse intestinal ganglioneuromatosis
7	53	M	GI bleeding	Colonic Schwann cell hamartoma
8	45	F	None	Gastric SEL, gastric fundic gland polyp, colorectal polyp
9	19	F	Nausea	Esophageal polyp
10	62	M	None	Gastric hyperplastic polyp
11	65	M	None	Colorectal polyp
12	48	F	Anemia	Colorectal adenoma
13	62	M	None	Esophageal polyp, colorectal adenoma

Abbreviations: GI, gastrointestinal; GIST, gastrointestinal stromal tumor; SEL, subepithelial lesion.

**FIGURE 3:**
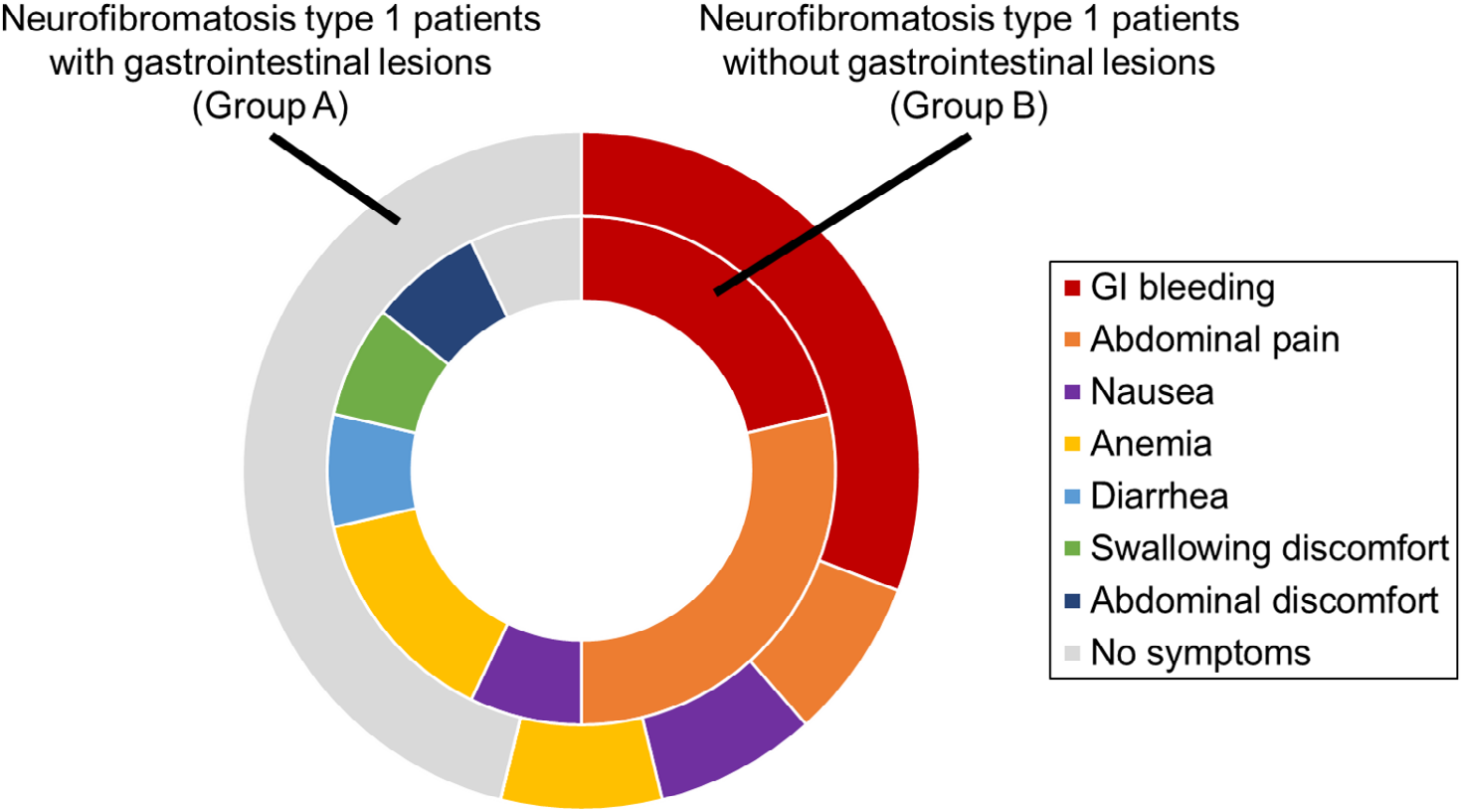
A donut plot illustrating the symptoms that prompted endoscopic examinations. Gastrointestinal lesions were detected during endoscopy in 13 patients with NF1 (Group A). In contrast, 14 patients with NF1 had no gastrointestinal tract lesions (Group B). The illustration indicates the symptoms that were the reasons for endoscopic examination in both groups. GI, gastrointestinal; NF1, neurofibromatosis type 1.

Figure 3 shows a donut plot illustrating the presenting symptoms that led to the endoscopic examinations in Groups A and B. In Group A, the most common finding was the absence of any symptoms at the time of endoscopy (six cases, 46%). These patients underwent endoscopic examinations either for screening purposes or as part of routine annual checkups. Four patients (31%) underwent endoscopy due to gastrointestinal bleeding. Other reasons, each accounting for one case (8%), included abdominal pain, nausea, and anemia. Conversely, only one patient in Group B was asymptomatic and underwent endoscopy for screening purposes. Reasons for endoscopy in Group B included abdominal pain (*n* = 4, 27%), gastrointestinal bleeding (*n* = 3, 20%), anemia (*n* = 2, 13%), diarrhea (*n* = 1, 7%), nausea (*n* = 1, 7%), swallowing discomfort (*n* = 1, 7%), and abdominal discomfort (*n* = 1, 7%). Group A had a significantly higher proportion of asymptomatic patients compared to Group B (*p* = 0.032).

**FIGURE 4:**
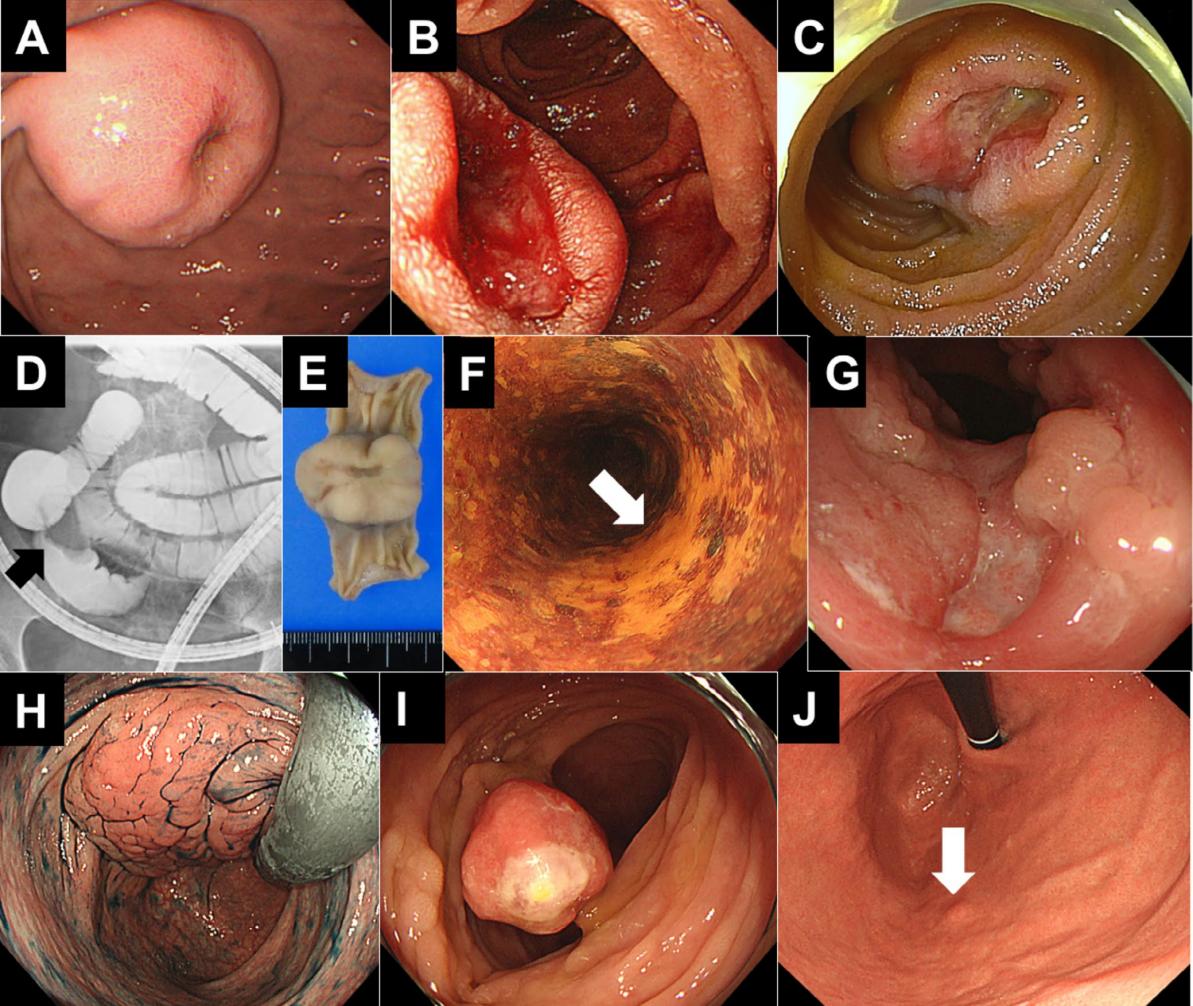
Endoscopic images of gastrointestinal lesions detected in patients with NF1. A gastrointestinal stromal tumor (GIST) in the stomach (A, Case 1). A GIST in the duodenum (B, Case 2). One of the jejunal GISTs (C, Case 3). Contrast imaging reveals a translucent lesion in the ileum (D, Case 4). The resected ileal lesion shows an ulcerative tumor (E). One patient (Case 5) had early esophageal cancer (F) and diffuse ganglioneuromatosis (G) metachronously. Diffuse ganglioneuromatosis is presented as a large, tumorous lesion in the rectum in another patient (H, Case 6). A Schwann cell hamartoma in the colon (I, Case 7). A small subepithelial lesion in the stomach (J, Case 8).

Gastrointestinal lesions identified in Group A patients included colorectal polyp (including adenoma, *n* = 6, 26%), GIST (*n* = 4, 17%), subepithelial lesion (*n* = 3, 13%), gastric fundic gland polyp (*n* = 3, 13%), diffuse intestinal ganglioneuromatosis (*n* = 2, 9%), esophageal polyp (*n* = 2, 9%), Schwann cell hamartoma (*n* = 1, 4%), esophageal cancer (*n* = 1, 4%), and gastric hyperplastic polyp (*n* = 1, 4%). Figure 4 presents representative endoscopic images of various gastrointestinal lesions identified in patients with NF1.

**FIGURE 5:**
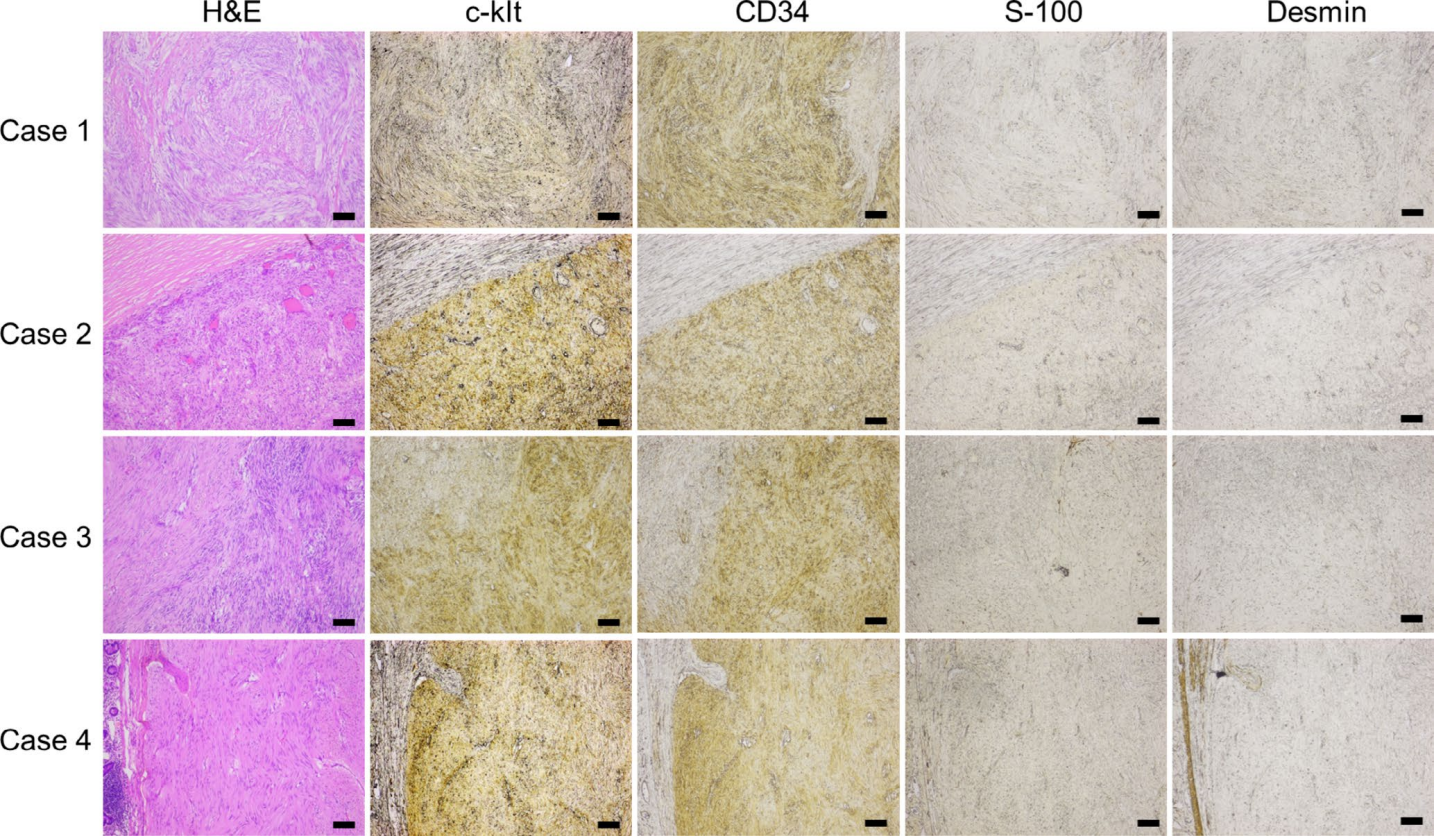
Immunostaining results of the GIST lesions from the patients with neurofibromatosis type 1. Gastrointestinal tissues from all four patients with neurofibromatosis type 1 included in this study were immunostained using c‐kit, CD34, S‐100, and Desmin antibodies. Representative staining results are shown along with the corresponding lesions stained with hematoxylin and eosin. Scale bar, 100 μm.

Table 2 summarizes the clinical characteristics of patients with GISTs. All four patients were male, aged between 44 and 76 years. Two patients presented with solitary GISTs located in the stomach (Case 1, Figure 4A) and duodenum (Case 2, Figure 4B), respectively. The remaining two patients had multiple GISTs in the small intestine (Case 3, jejunum; Case 4, both the jejunum and ileum). Peranal double‐balloon enteroscopy failed to detect the tumor in Case 4; however, contrast imaging revealed a translucent lesion in the ileum (Figure 4D). The resected specimen from this case showed an ulcerative tumor in the ileum (Figure 4E). All four GIST cases exhibited ulcer formation (Figure 4A–C). Immunostaining results were positive for c‐kit and CD34, but negative for S‐100 and desmin in all patients (Figure 5). Genetic analysis for mutations in the *KIT* gene (Case 1) and both the *KIT* and *PDGFRA* genes (Case 4) did not identify any abnormalities. All patients underwent surgical resection and have remained recurrence‐free for a follow‐up period ranging from 0.1 to 7.5 years.

**TABLE 2:** Characteristics of GISTs detected in patients with neurofibromatosis type 1.

Case no.	Age	Sex	Organ	Size (mm)	Nuclear fission (50 HPF)	Immunostaining for c‐kit	Mutation of the *KIT* gene	Mutation of *PDGFRA* gene	Outcome	Follow‐up period (years)
1	53	M	Stomach	38	6	Positive	Negative	NA	Alive	7.5
2	44	M	Duodenum	20	7	Positive	NA	NA	Alive	5.8
3	54	M	Jejunum	34^a^	< 5	Positive	NA	NA	Alive	6.1
4	76	M	Jejunum to ileum	30^a^	1	Positive	Negative	Negative	Alive	0.1

Abbreviations: HPF, high‐power field; NA, not available.

^a^ Owing to the presence of multiple lesions, the diameter of the largest tumor is indicated.

A 57‐year‐old male (Case 5) underwent endoscopic submucosal dissection for early esophageal cancer (Figure 4F). Later, at the age of 67, he underwent a total colectomy due to refractory ulcerative lesions caused by diffuse ganglioneuromatosis (Figure 4G). The patient's clinical course has been previously reported [7].

A 27‐year‐old male (Case 6) with rectal wall thickening on whole‐body MRI had a subsequent colonoscopy revealing a large tumorous lesion in the rectum (Figure 4H). Biopsy confirmed the diagnosis of diffuse ganglioneuromatosis. Although surgical resection was recommended, the asymptomatic patient opted for observation.

A 53‐year‐old male (Case 7) presenting with visible blood in the stool underwent a colonoscopy, which revealed multiple colonic polyps. One ulcerated pedunculated polyp (Figure 4I) was endoscopically removed and diagnosed as a Schwann cell hamartoma.

Figure 4J shows a small subepithelial lesion in the stomach (Case 8).

## 4 Discussion

We reviewed 27 patients with NF1 who underwent endoscopic examination and identified various neoplastic lesions, including four patients with GISTs (17%), three with gastrointestinal subepithelial lesions (13%), two with diffuse intestinal ganglioneuromatosis (9%), one with colonic Schwann cell hamartoma (4%), and one with esophageal cancer (4%). All GISTs and one case of diffuse intestinal ganglioneuromatosis were surgically resected. These findings highlight the potential for gastrointestinal complications in NF1 patients, particularly GISTs.

In patients with NF1, the prevalence of GISTs is relatively high, ranging from 4% to 25% [8, 9, 10, 11, 12, 13, 14]. A study conducted in Japan revealed that CT screening of 95 patients with NF1 identified six cases of GIST, indicating a prevalence rate of approximately 6.3 per 100 patients with NF1 [15]. According to Japanese guidelines, asymptomatic patients with NF1 should not undergo screening for neoplastic lesions, including GISTs [16]. This recommendation is based on reports suggesting that there is no difference in treatment outcomes between patients treated after symptom onset and those treated after neoplasms are detected by screening [17]. Therefore, the guidelines recommend consulting gastroenterologists for a thorough examination when gastrointestinal symptoms such as melena or abdominal pain appear in patients with NF1. Similarly, tumor surveillance guidelines for individuals with NF1 issued by the European Reference Network on Genetic Tumor Risk Syndromes (ERN GENTURIS) state that GIST surveillance should be performed based on the clinical suspicion of symptoms [18]. However, some opinions advocate CT screening in adult patients with NF1 over 30 years of age [15]. Others have recommended that patients with known germline mutations undergo baseline CT of the abdomen/pelvis and esophagogastroduodenoscopy with endoscopic ultrasound upon identification as carriers of NF1 [19].

NF1‐associated GISTs are typically indolent, with a low mitotic rate and favorable prognosis [14]. Unlike sporadic GISTs, those in NF1 patients often lack *KIT* and *PDGFRA* gene mutations, rendering the drug imatinib ineffective. This makes surgical resection the primary treatment option. However, the postoperative recurrence rate of GIST in NF1 patients (18.2%) is not significantly lower compared to non‐NF1 patients (24.9%) [15]. A study focusing on death certificates of NF1 patients revealed GISTs as the leading cause of mortality in seven out of 232 deceased individuals [20]. These findings highlight the potential benefit of early GIST detection and surgical management in NF1 patients, potentially improving prognosis.

Here, approximately 50% of the patients in group A were asymptomatic during endoscopy, which highlights the potential presence of undetected gastrointestinal lesions in NF1 patients. Additionally, all patients who required surgery for gastrointestinal tumors were aged ≥ 40 years. Although most GIST cases in our study were symptomatic, NF1‐associated GISTs are resistant to imatinib, making early detection and curative resection particularly important for NF1 patients. Therefore, we propose that NF1 patients aged ≥ 40 years must undergo at least one esophagogastroduodenoscopy and colonoscopy, along with imaging modalities, such as CT and MRI, to screen for gastrointestinal lesions, with the need for further follow‐up determined based on individual risk factors. Although no established guidelines currently exist for screening asymptomatic NF1 patients, a follow‐up interval of 2–5 years may be reasonable. A limitation of this study is that only 27 of the 225 NF1 patients underwent endoscopic examinations, and the findings are based on a relatively small dataset. Thus, large‐scale prospective studies are necessary to refine screening recommendations. Given that NF1 patients frequently seek care from specialists in dermatology, neurology, orthopedics, and plastic surgery, raising awareness among physicians across specialties regarding the potential for gastrointestinal neoplasms in NF1 is crucial.

We also reviewed patients with NF2, none of whom underwent endoscopic examinations. NF2 is characterized by bilateral vestibular schwannomas and is caused by mutations in the *NF2* gene [21, 22, 23]. While distinct from NF1, an association between NF2 and gastrointestinal disorders has not been reported.

We identified various gastrointestinal lesions, including GISTs, in 13 of 27 patients with NF1 who underwent endoscopy. Notably, half of these patients with lesions were asymptomatic, highlighting the potential for early detection. All patients requiring surgical intervention for gastrointestinal tumors were 40 years of age or older. Although validation through large‐scale studies is required eventually, surveillance endoscopy, combined with imaging techniques, such as CT and MRI, may be beneficial for NF1 patients aged ≥ 40 years in screening for gastrointestinal lesions. Given that NF1 patients consult various specialists, educating physicians across specialties about the potential for gastrointestinal complications is crucial.

## Ethics Statement

This study received approval from the Ethics Committee of Okayama University Hospital. Due to the retrospective nature of the study and the use of anonymized clinical data, the requirement for written informed consent was waived.

## Conflicts of Interest

The authors declare no conflicts of interest.

## Data Availability

The data that support the findings of this study are available on request from the corresponding author.
